# Physiological and Molecular Mechanisms of Nano-Hydroxyapatite (nHAP) in Regulating Chilling Tolerance of Cucumber Seedlings

**DOI:** 10.3390/ijms27167358

**Published:** 2026-08-17

**Authors:** Wajid Anwar, Rui-Heng Cai, Ting Pang, Yungui Li, Dissanayakalage D. N. V. Dissanayaka, Yun-Song Lai

**Affiliations:** 1 College of Horticulture, Sichuan Agricultural University, Chengdu 611130, China; 2 School of Environment and Resources, Southwest University of Science and Technology, Mianyang 621010, China

**Keywords:** nano-fertilizer, cold stress, oxidative stress, RNA-seq, plant physiological indices

## Abstract

Xishuangbanna (XIS) cucumber (*Cucumis sativus*) originated from low-altitude southwest China and shows extreme cold sensitivity. Nano-hydroxyapatite (nHAP), known for its high bioavailability and surface reactivity relative to bulk HAP, was applied to enhance the cold tolerance of XIS seedlings. We optimized fertilization timing and fertilizer concentration. By determining physiological parameters alongside gene expression profiling and transcriptomic analysis, we preliminarily dissected the physiological and molecular mechanisms underlying nHAP-enhanced chilling tolerance. nHAP application preserved free and bound water even at 24 h into the cold-stress treatment, as detected by nuclear magnetic resonance (NMR) and magnetic resonance imaging (MRI). Exposure to cold stress caused a chilling injury index (CII) of 73.33%, while foliar spraying of nHAP at gradient concentration reduced CII values by 9.09–54.55%. At the same time, electrolyte leakage and malondialdehyde content were decreased by 6.68–61.41% and 12.92–67.16% respectively; chlorophyll content increased by 0.01–73.42%; SOD, POD, and soluble protein content increased by 10.54–161.53%, 16.50–154.33%, and 2.03–63.26%, respectively. Soil application of nHAP showed a similar but weaker effect than foliar spraying on alleviating chilling injury, and the optimal concentration was 1000 mg/L. Quantitative Real-Time PCR (qRT-PCR) revealed that foliar spraying of nHAP upregulated the gene expression of Superoxide dismutase genes (*CsCu/ZnSOD* and *CsMnSOD*) and major facility superfamily genes (*CsSPX-MFS1* and *CsSPX-MFS2*) in the cold treatment. We then profiled the transcriptome changes in seedling leaves during the cold treatment after foliar spraying of nHAP. Without nHAP application, cold stress resulted in a total of 6795 differentially expressed genes (DEGs), which were functionally enriched in plant–pathogen interaction and plant hormone signal transduction pathways. Under nHAP application, cold stress only caused 776 DEGs, which were functionally enriched in plant hormone signal transduction and galactose metabolism. These findings suggest that nHAP could improve the cold tolerance of cucumber seedlings through boosting antioxidant activity and plant hormone signaling pathways.

## 1. Introduction

Cucumbers are documented to be one of the oldest domesticated vegetables [1]. Xishuangbannanesis (XIS) cucumber (*Cucumis sativus* L. *var. Xishuangbannesis Qi et Yuan*) is a semi-wild subspecies that was discovered in the 1980s in the Xishuangbanna prefecture of China [2]. It exhibits several unique traits, such as sexual sensitivity to photoperiod conditions [3], flowering under short-day conditions [4], hypocotyl tolerance to low light intensity [5], and extreme heat tolerance and high cold sensitivity in germplasm evaluation [6,7].

Cold stress negatively affects cucumber growth and development by reducing germination rates, causing leaf wilting and yellowing at the seedling stage, decreasing fruit set, and potentially leading to plant death. Plants employ cold tolerance mechanisms at multiple levels, including physiological, biochemical, transcriptional, and ion homeostatic, to stabilize cellular functions via enhanced synthesis of proteins, carbohydrates, and soluble sugars such as sucrose [8]. Physiological mechanisms include alterations in the plant cell membrane and antioxidant system. Cold stress induces lipid peroxidation by increasing the level of malondialdehyde (MDA) [9]. A deleterious effect of cold stress on plant cells is the production of reactive oxygen species (ROS), such as superoxide anions (O_2_^−^) and hydrogen peroxide (H_2_O_2_), causing cellular damage [10]. To protect plants from the adverse effects of ROS, both enzymatic and non-enzymatic antioxidants are activated [11].

Low-temperature stress triggers comprehensive gene responses in plants, involving signal transduction, transcription regulation, and functional protein synthesis. Key responses include the activation of the ICE-CBF-COR cascade, where ICE induces CBF transcription factors, which further activate COR genes [12]. Genes related to cold tolerance mainly involve CBF/DREB1, ICE1, and the COR family, which encode osmoregulatory proteins, antifreeze proteins, and antioxidant enzymes [13]. Additionally, MAPK and Ca^2+^ signaling-related genes, as well as ROS scavenging genes, contribute to enhancing cold tolerance by stabilizing cell membranes and reducing oxidative damage [14]. Beyond these major signaling pathways, phytohormones also play a critical role in regulating chilling tolerance. Salicylic acid has been shown to enhance chilling tolerance in cucumber through the CsNPR1–CsICE1 pathway, linking hormone signaling directly to the ICE-CBF-COR cascade [15]. Other hormones, including abscisic acid (ABA), jasmonic acid (JA), and ethylene, are similarly implicated in cold-stress responses, modulating antioxidant defense, stomatal regulation, and stress-responsive gene expression [16,17].

Hydroxyapatite (HAP, Ca_10_(PO_4_)_6_(OH)_2_) is a biocompatible calcium phosphate mineral, widely used as a slow-release phosphorus (P) fertilizer in agriculture. Naturally occurring hydroxyapatite derived from phosphate rock is commonly used as a fertilizer. Compared to bulk HAP, nano-HAP (nHAP) offers distinct advantages. Its tiny particle size (20–100 nm) provides a larger specific surface area, enables controlled nutrient release, reduces P leaching, and improves use efficiency [18,19]. Importantly, phosphorus in the form of nHAP exhibits enhanced solubility, which can overcome the limited solubility of HAP [20]. nHAP can enhance phosphorus availability and improve plant growth in various vegetable crops, including tomato [21], lettuce [22], and bell pepper [23].

Some studies have examined the role of nHAP in mitigating abiotic stress in vegetables. nHAP effectively reduced cadmium (Cd) toxicity in soil by decreasing cadmium uptake and increasing biomass accumulation in pakchoi [24]. In addition, nHAP can enhance antioxidant enzyme activity and reduce MDA content in plants under oxidative stress, thereby minimizing damage to plant tissues [23]. Most studies on nanoparticle- and nanofertilizer-mediated stress tolerance have focused on drought, salinity, or heavy metal toxicity, while the potential of nHAP in alleviating cold/chilling stress remains largely unexplored [25].

Cucumber is specifically vulnerable to low temperature stress, which severely disrupts growth and development by inducing excessive ROS production [26]. Given nHAP’s demonstrated capacity to enhance antioxidant defense and reduce oxidative damage under other abiotic stresses, we hypothesized that nHAP application may enhance the antioxidant system in cucumber under cold stress, thereby maintaining cellular homeostasis and reducing stress-induced damage. This study aims to explore the potential of nHAP in enhancing cold-stress tolerance in XIS cucumbers.

## 2. Results

### 2.1. Fertilizer Efficiency Comparison Between nHAP and Conventional HAP

**Figure 1:**
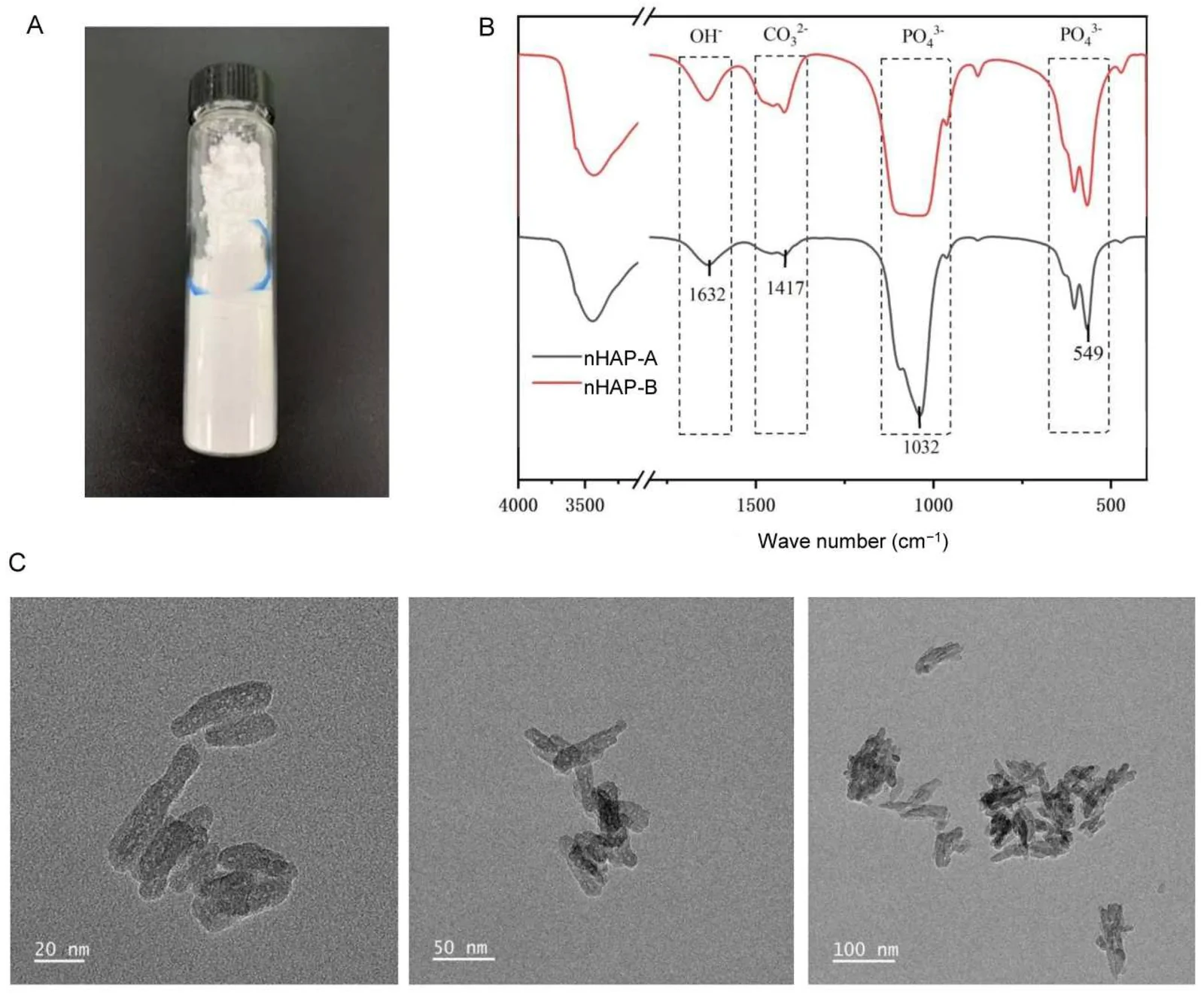
Physical characterization of prepared nHAP. (**A**) Appearance of final nHAP product. (**B**) Fourier transform infrared spectra of nHAP pellet mixed with potassium bromide. nHAP-A, nHAP synthesized via wet chemical precipitation method; nHAP-B, nHAP synthesized via ultrasonication. (**C**) Nanoparticle size of nHAP under a microscope.

The nHAP powder synthesized via wet chemical precipitation exhibits a pure white hue upon thorough drying (Figure 1A). nHAP was mixed with potassium bromide powder to make a thin pellet after drying and tableting to detect the characteristic Fourier transform infrared spectra (FTIR) of HAP (Figure 1B). The FTIR spectra showed characteristic absorption bands corresponding to OH^−^ (1632 cm^−1^), CO_3_^2−^ (1417 cm^−1^), and PO_4_^3−^ (1032 and 549 cm^−1^) functional groups, confirming the successful synthesis of hydroxyapatite. Finally, the nanoscale particle size of nHAP was confirmed via microscopic examination (Figure 1C). Elongated and rod-shaped nanoparticles were approximately 5–10 nm in width and 20–60 nm in length, confirming that the synthesized material falls within the nanoscale size range.

**Figure 2:**
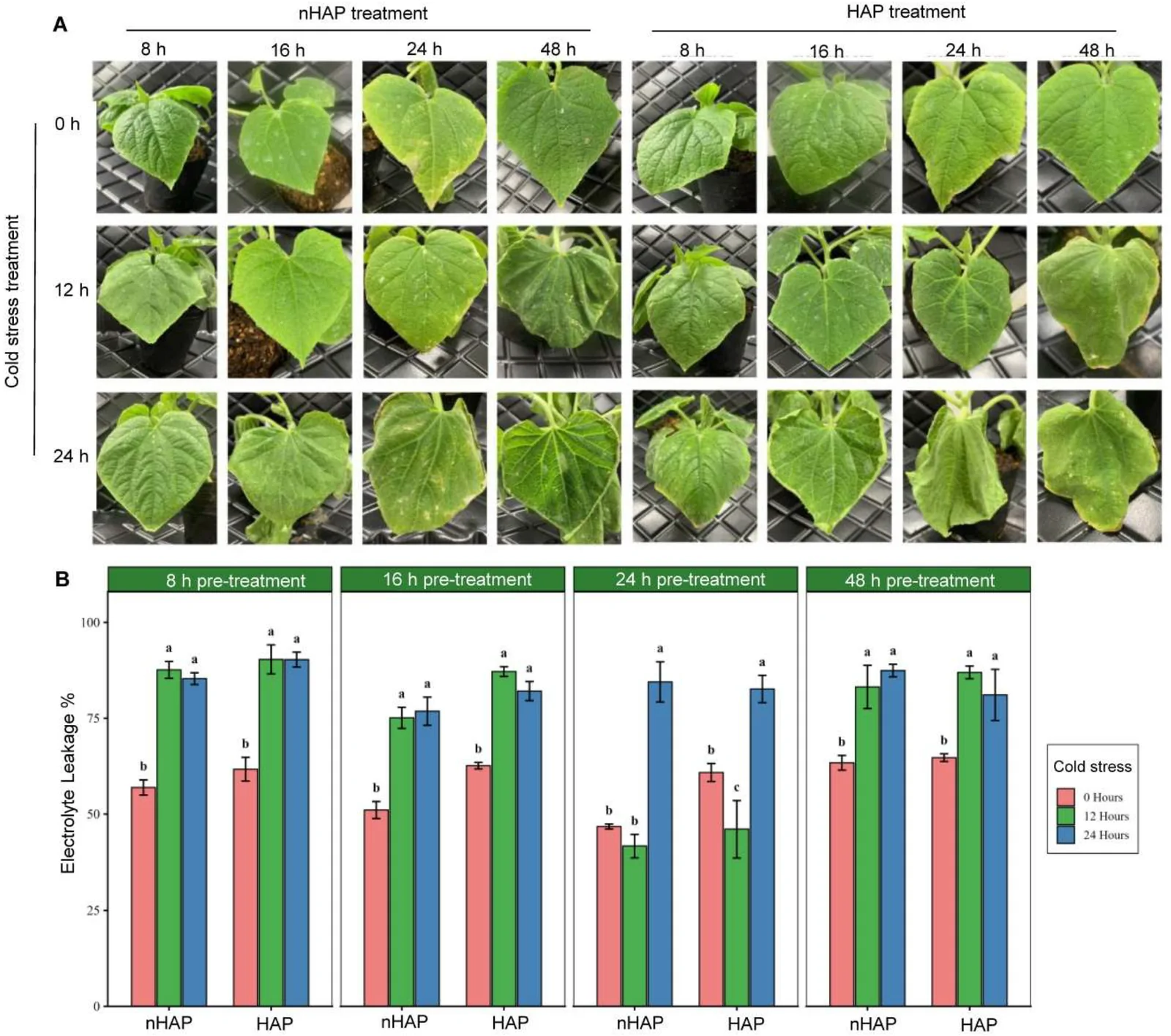
Nano-fertilizers enhance the low-temperature tolerance of cucumber seedlings. (**A**) Appearance of cucumber seedling in the cold treatment. (**B**) Electrolyte leakage in the cold treatment under nHAP and HAP application. nHAP was applied at 8 h, 16 h, 24 h and 48 h before cold stress. Vertical bars show SEs of means (*n* = 3). Letters above bars denote significant differences among treatments (Tukey–Kramer HSD post hoc test with Benjamini–Hochberg FDR correction, *p* < 0.05).

Taking HAP as the control, we evaluated the fertilizer effect of nHAP under cold treatment through foliar application (Figure 2). Seedlings sprayed with nHAP had healthier leaves than those treated with HAP during cold treatment, even 48 h after fertilizer application (Figure 2A). This indicates that nHAP performed better than HAP, not only in term of fertilizer efficiency but also in the persistence of its effect. According to the EL values (Figure 2B), the greatest difference between nHAP and HAP occurred at 12 h during the cold treatment that started 24 h after the fertilizer application. For nHAP application, the symptoms of chilling injury did not deteriorate significantly within 12 h in the case of nHAP application.

### 2.2. Evaluation of Cold Injury Using NMR and MRI

**Figure 3:**
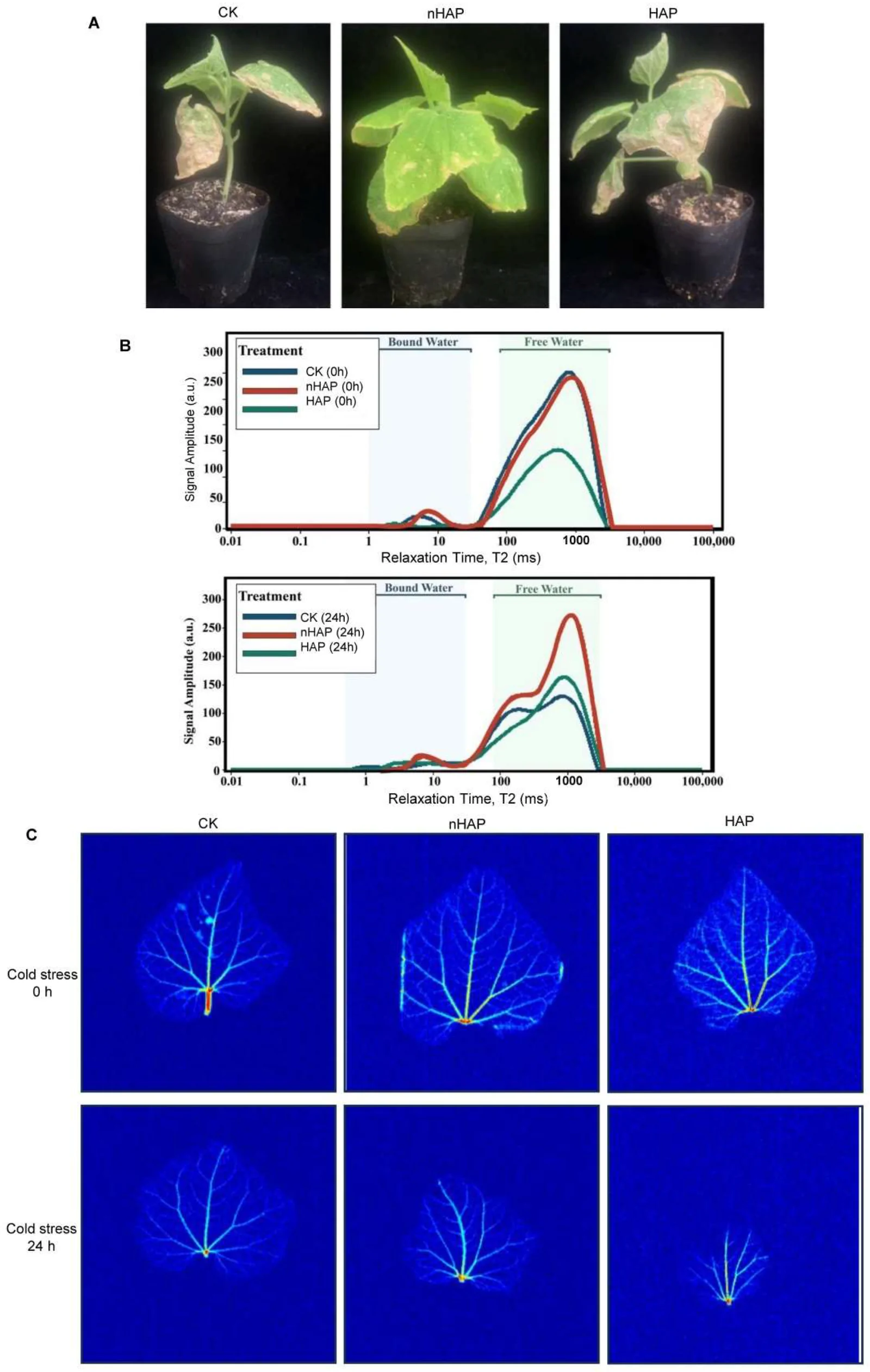
Evaluation of cold injury using low-field NMR and MRI. (**A**) Seedlings after cold treatment for 24 h were used in MRI assays. (**B**) T2 relaxation spectrum of the seedling leaves before (**top**) and after (**bottom**) cold treatment. (**C**) Biological water status after 24 h in the cold treatment was visualized by MRI. Color scale indicates signal intensity, blue (low) and red/yellow (high).

Cold injury was evaluated based on biological water, which was quantified and visualized using LF-NMR and MRI (Figure 3). Seedlings treated with fertilizer 24 h earlier were subjected to cold stress. nHAP-treated plants maintained a healthier phenotype with green leaves compared to the control and HAP-treated plants (Figure 3A). As visualized by MRI, 24h cold treatment negatively affected biological water in all samples, but nHAP plants had brighter signals in the veins, indicating healthier water flow in vascular bundles and therefore had better metabolic activity, under cold stress (Figure 3B). To quantify the variations in biological water, we extracted the signal intensities corresponding to different water fractions (Figure 3C). Before the cold treatment, plants supplied with nHAP exhibited the highest bound water as well as free water. After the cold treatment, all the plants showed disturbed biological water status, but nHAP plants were the least affected. Although free water detected at 750 ms was slightly affected, the contents of other biological water fractions were not significantly affected. Bound water inside the cell reflects less ice crystal formation under cold stress and therefore indicates relatively healthy biological structure in nHAP plants, and free water means metabolic activity.

### 2.3. Influence of nHAP Concentration and Application Method

**Figure 4:**
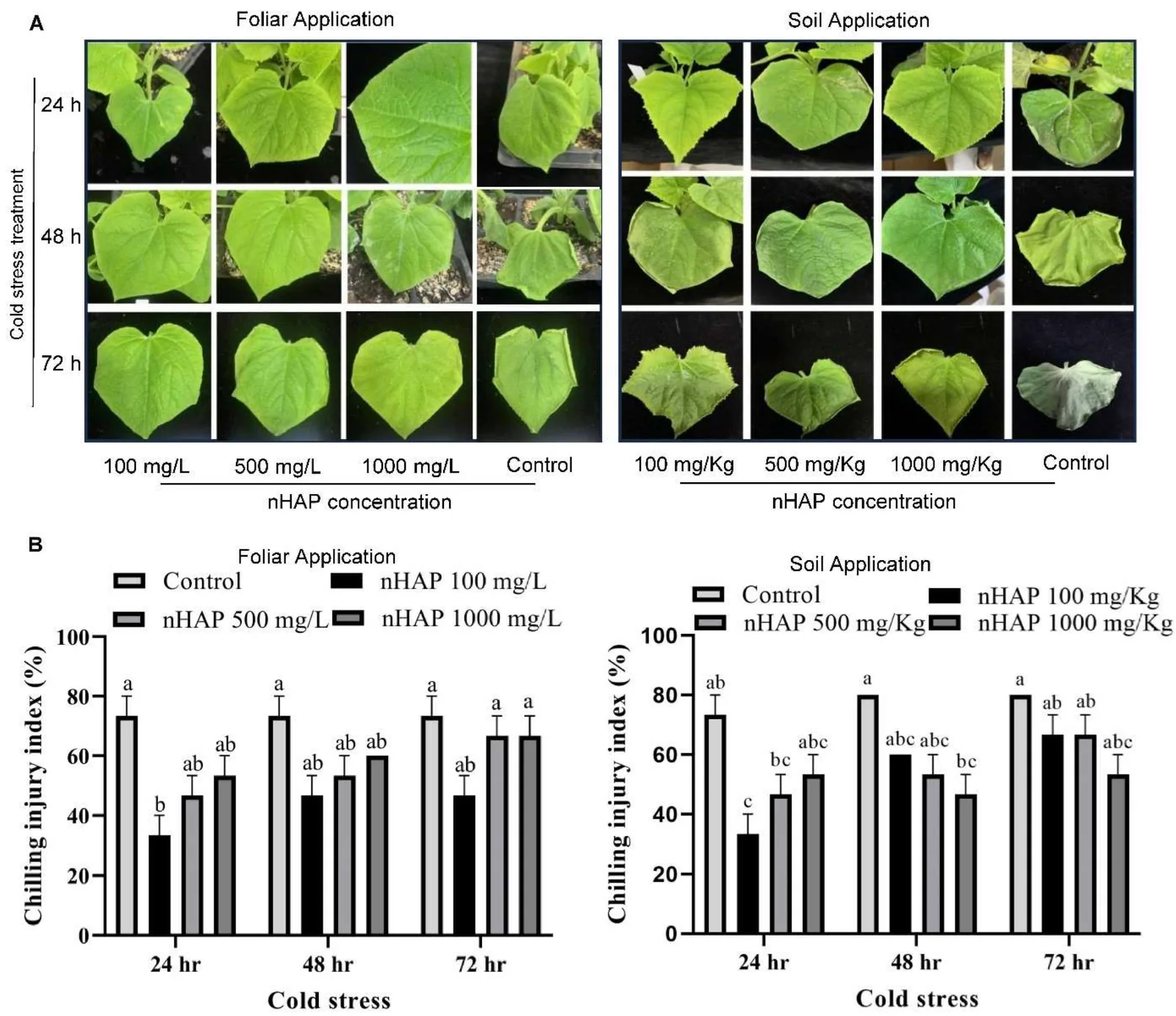
Effects of concentration and nHAP application method on cold tolerance. (**A**) Appearance of cucumber seedlings in the cold treatment. (**B**) Chilling injury index. Left, foliar application; right, soil application. Measurements were taken at 24 h, 48 h and 72 h during the cold stress. The cold treatment started 24 h after the nHAP application. Vertical bars show SEs of means (*n* = 3). Letters above bars denote significant differences among treatments (Tukey–Kramer HSD post hoc test with Benjamini–Hochberg FDR correction, *p* < 0.05).

We established different concentration gradients and application methods to optimize fertilization regimes for nHAP (Figure 4). Clearly, both foliar and soil application of nHAP significantly alleviated cold injury and delayed the deterioration in the CII. The control plants exhibited sever chilling injury within 24 h, whereas seedlings treated with nHAP, either through foliar application or soil application, showed a comparative CII only after 72 h. After 72 h of cold stress, foliar application with a concentration of 100 mg/L reduced CII by 36.36%, while the overall nHAP gradient concentration reduced CII by 9.09–54.55% during all cold-stress time points. Soil application reduced CII by 16.67–33.33% after 72 h of cold stress. Foliar spraying outperformed root drenching. At the same concentration, foliar application generally resulted in lower chilling injury than soil application. For foliar application, cold injury increased with higher nHAP concentration; in contrast, a higher concentration of nHAP resulted in lower cold injury at 48 h and 72 h in the cold treatment. This indicates that only high concentrations of nHAP are more effective when applied to the roots but act more slowly. In summary, the overall rankings were nHAP100 > nHAP500 > nHAP1000 > control by foliar application and nHAP1000 > nHAP500 > nHAP100 > control by soil application. The best performance was observed in the foliar application at nHAP concentrations of 100 mg/L and 1000 mg/L.

### 2.4. Effects of nHAP on Plant Physiological Indices

We evaluated the major plant physiological indices, including EL, MDA content, soluble protein, chlorophyll content, and carotenoid content, to assess the physiological effects of nHAP.

**Figure 5:**
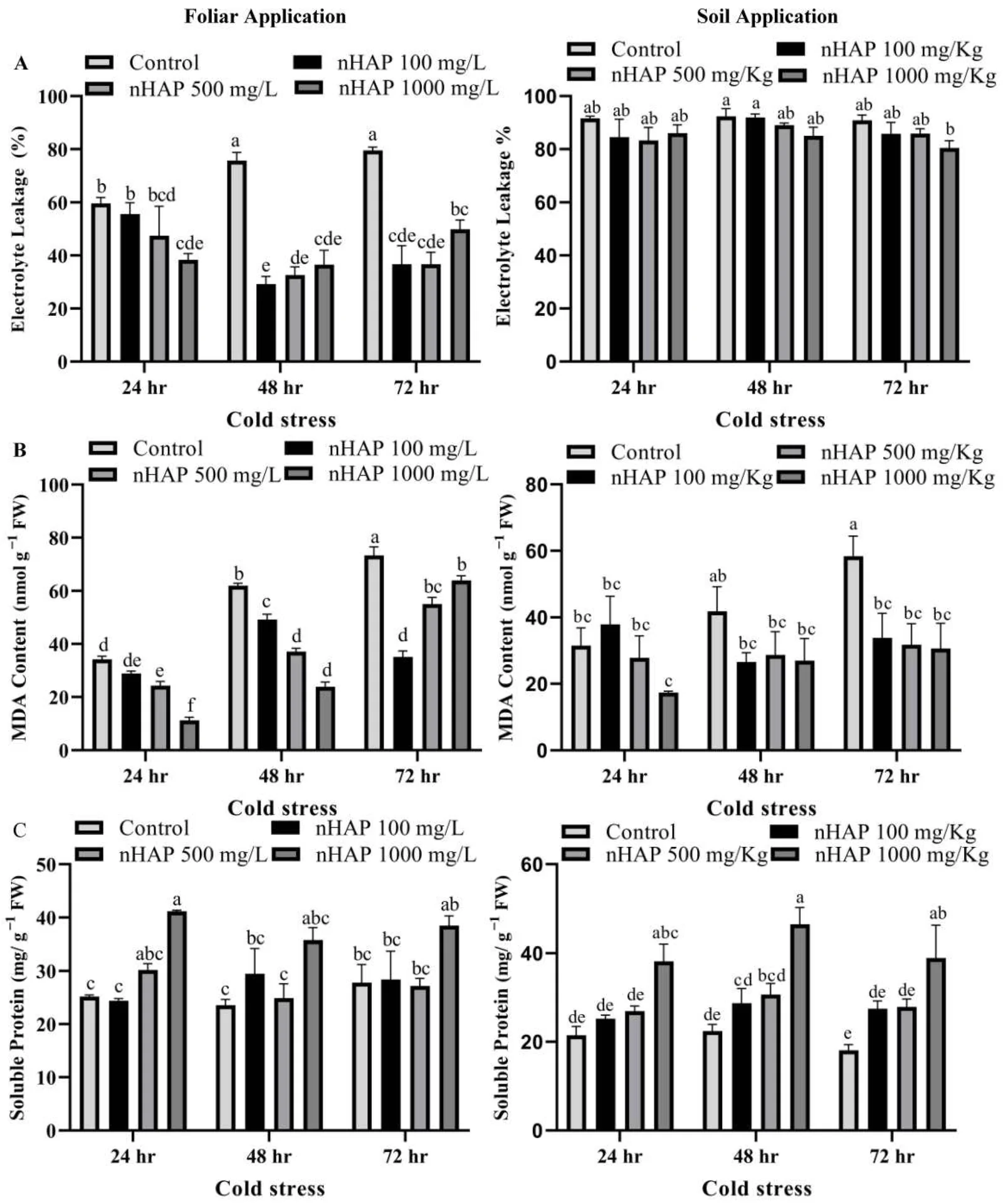
Effects of concentration and nHAP application method on physiological indices during the cold treatment. (**A**) Relative electrolyte leakage %. (**B**) Malondialdehyde content (MDA). (**C**) Soluble protein. Left, foliar application; right, soil application. Measurements were taken at 24 h, 48 h and 72 h during the cold stress. Vertical bars show SEs of means (*n* = 3). Letters above bars denote significant differences among treatments (Tukey–Kramer HSD post hoc test with Benjamini–Hochberg FDR correction, *p* < 0.05).

High EL indicates cell membrane damage and chilling injury (Figure 5A). Under cold treatment, the EL values increased rapidly to 59.64% within 24 h and reached the peak of 79.51% within 72 h. Soil application of nHAP had little effect in terms of reducing EL, except at 72 h when nHAP at 1000 mg/kg reduced EL by 11.47%. In contrast, foliar application significantly decreased EL values. At 24 h of cold treatment, higher concentrations of nHAP showed a better effect, but at 48 h and 72 h the lower concentrations were more effective. This implies that higher concentrations produce a faster fertilizer effect.

MDA content gradually increased during cold treatment but was significantly suppressed by nHAP application (Figure 5B). In the foliar application, higher concentrations of nHAP performed better, although 1000 mg/Lwas less effective at 72 h. Overall, foliar application of nHAP reduced MDA content at nHAP 500 and 1000 mg/L. In the soil application, MDA content was significantly decreased by higher concentrations of nHAP. Again, a higher concentration causes a faster fertilizer effect in terms of MDA.

No significant changes in soluble protein content were observed during the cold treatment in the control (Figure 5C). Application of high concentrations of nHAP could significantly increase soluble protein, which may enhance plant resistance to cold stress. In the foliar application, treatment with nHAP at 1000 mg/L increased soluble protein by 63.24% at 24 h, 51.75% at 48 h and 38.28% at 72 h. In the soil application, treatment with 1000 mg/kg showed a 77.02% increment at 24 h, a 107.23% increment at 48 h, and a 115.20% increment at 72 h. These results indicate that nHAP can rapidly enhance soluble protein accumulation under cold stress.

**Figure 6:**
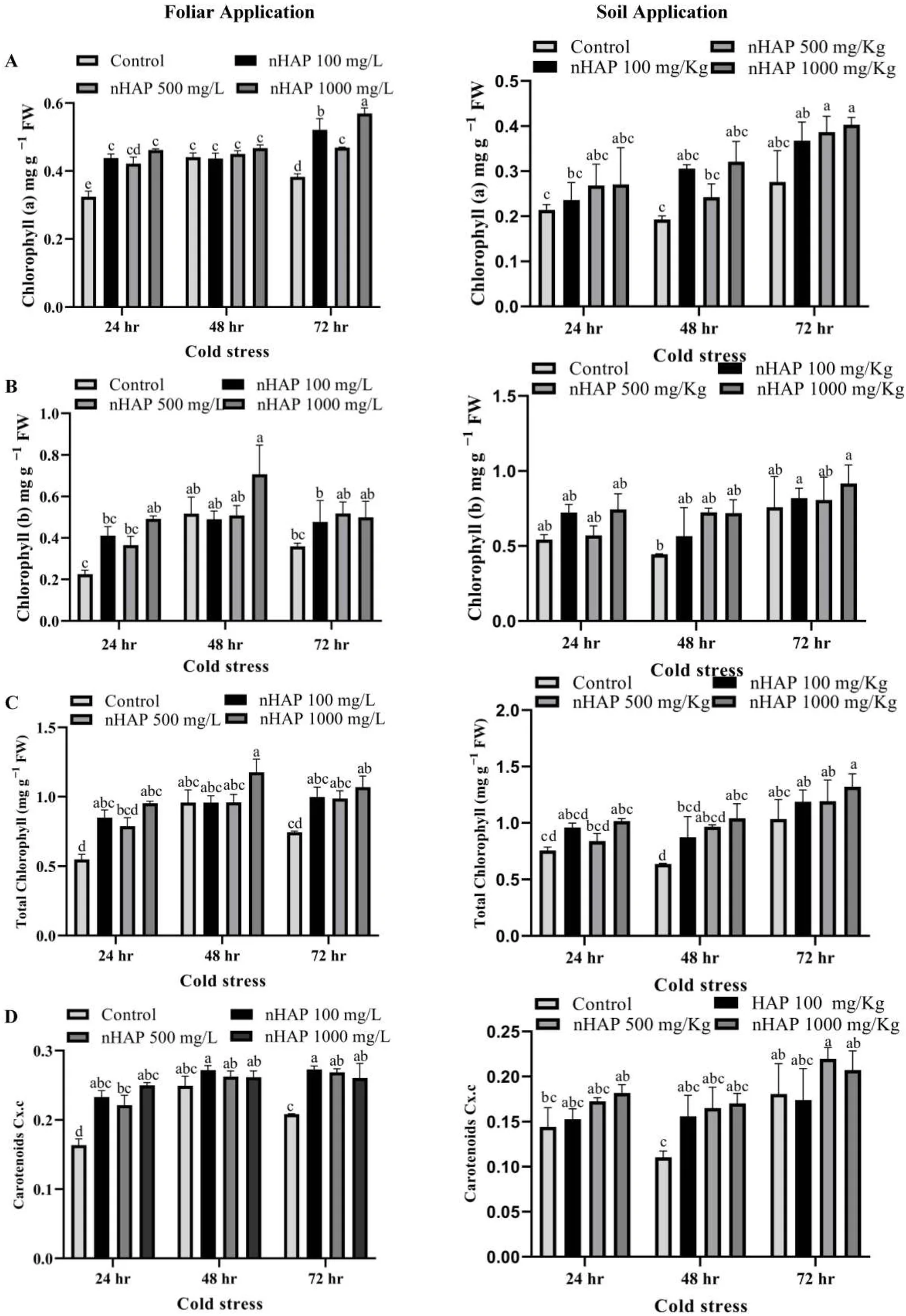
Effects of concentration and nHAP application method on pigments during the cold treatment. (**A**) Chlorophyll a (Chla). (**B**) Chlorophyll b (Chlb). (**C**) Total chlorophyll. (**D**) Total carotenoid concentration. Left, foliar application; right, soil application. Measurements were taken at 24 h, 48 h and 72 h during the cold stress. Vertical bars show SEs of means (*n* = 3). Letters above bars denote significant differences among treatments (Tukey–Kramer HSD post hoc test with Benjamini–Hochberg FDR correction < 0.05).

Regarding chlorophyll content, the control plants showed a slight increase, which was probably due to the water loss in leaves (Figure 6A–C). Clearly, nHAP application resulted in a higher chlorophyll content. For example, 1000 mg/L foliar application increased chlorophyll content by 43.96% after 72 h of cold stress. The promoting effect of nHAP on A under 72 h cold stress treatment was also verified via soil application. Generally, gradient concentrations of nHAP increased chlorophyll content by 0.01–73.42% across different treatments and time points. Chlorophyll b levels were higher at 24 h and 72 h in the foliar application, while total chlorophyll content increased at 24 h and 72 h in the foliar application and at 48 h in the soil application.

Similar to chlorophyll, another class of pigments, carotenoids, also showed fluctuating content in leaves during the cold treatment in the controls (Figure 6D). Foliar application slightly increased carotenoid content. In the foliar application, nHAP at 1000 mg/L led to an increase of 52.73% at 24 h and 5.01% at 48 h compared to the control. At 72 h, nHAP at 100 mg/L resulted in a 30.96% increment compared to the control. In the soil application, the carotenoid content showed a slight increase with nHAP 1000 mg/Kg as compared to the control.

### 2.5. Effects of nHAP on the Accumulation of O_2_^-^ and H_2_O_2_

**Figure 7:**
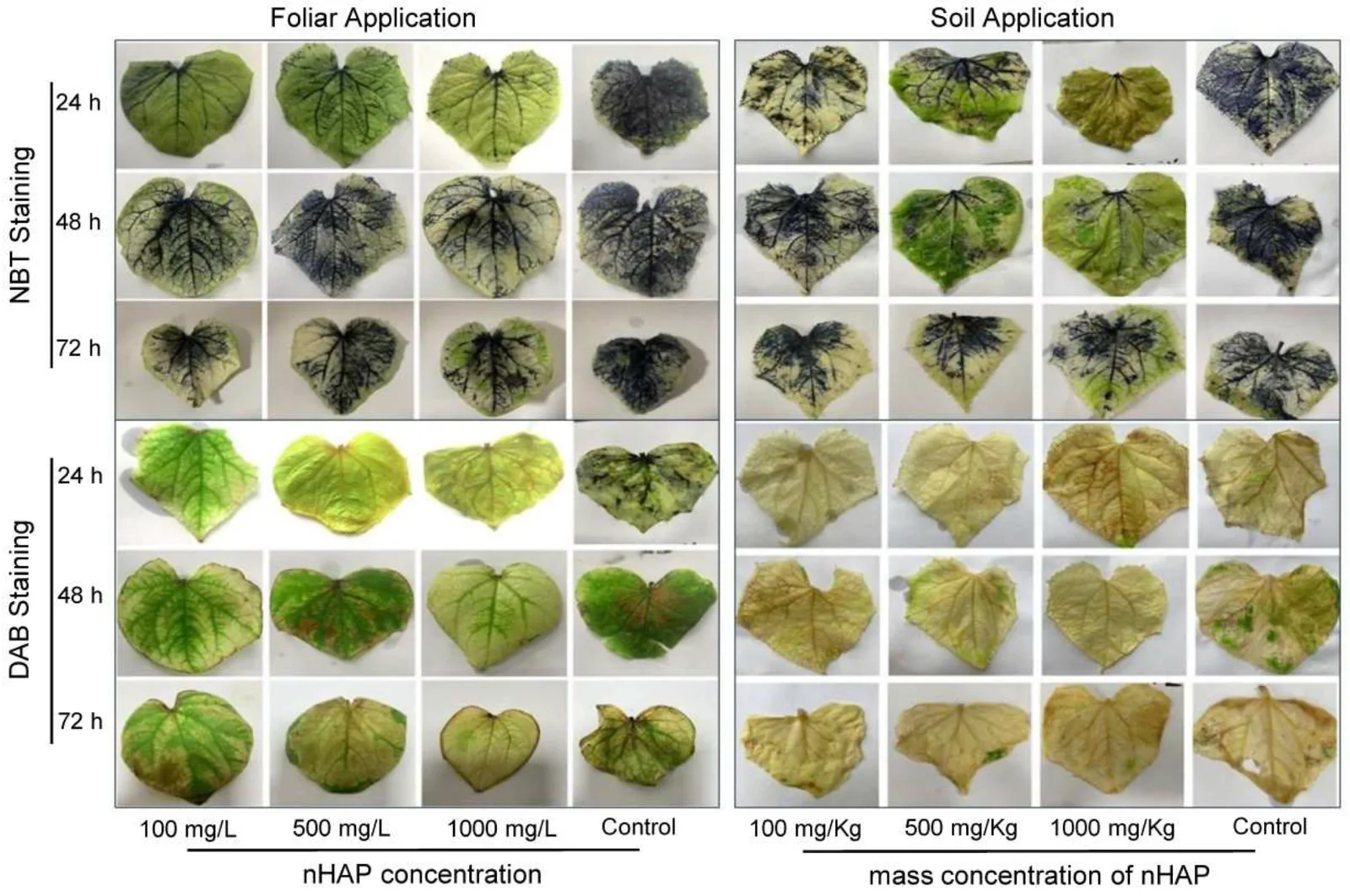
Effects of concentration and nHAP application method on ROS accumulation during the cold treatment. (**Left**) Foliar application. (**Right**) Soil application. Measurements were taken at 24 h, 48 h and 72 h during the cold stress.

O_2_^−^ and H_2_O_2_ are major ROS in plants. The accumulation causes oxidative damage under low-temperature stress. We quantified O_2_^−^ by NBT staining and H_2_O_2_ by DAB staining (Figure 7). Here, NBT staining offers fast and clear visualization of O_2_^−^ as a blue color, and DAB staining provides precise localization of H_2_O_2_ with stable brown deposits. NBT staining clearly showed that the blue coloration diffused from the veins into the mesophyll tissue, whereas DAB staining showed that H_2_O_2_ accumulation originated from both the veins and leaf margins, appearing as large spots. Basically, the content of O_2_^−^ and H_2_O_2_ were consistent with the results of the injury index. Control plants exhibited intense staining distributed across the entire leaf, while nHAP-treated plants showed only partial staining. Foliar application of nHAP resulted in fewer blue spots (indicating lower accumulation of O_2_^−^ and H_2_O_2_) than soil application at 24 h, indicating that nHAP fertilizer acts more rapidly when applied as a foliar spray. Regarding concentration gradients, there were no major differences among treatments in the foliar application, whereas higher concentrations performed better in the soil application, with 1000 mg/kg showing the greatest reduction in O_2_^−^ accumulation. Interestingly, foliar application was more effective than soil application in reducing H_2_O_2_ accumulation, as evidenced by DAB staining.

### 2.6. Effects of nHAP on Antioxidant Enzyme Activity

**Figure 8:**
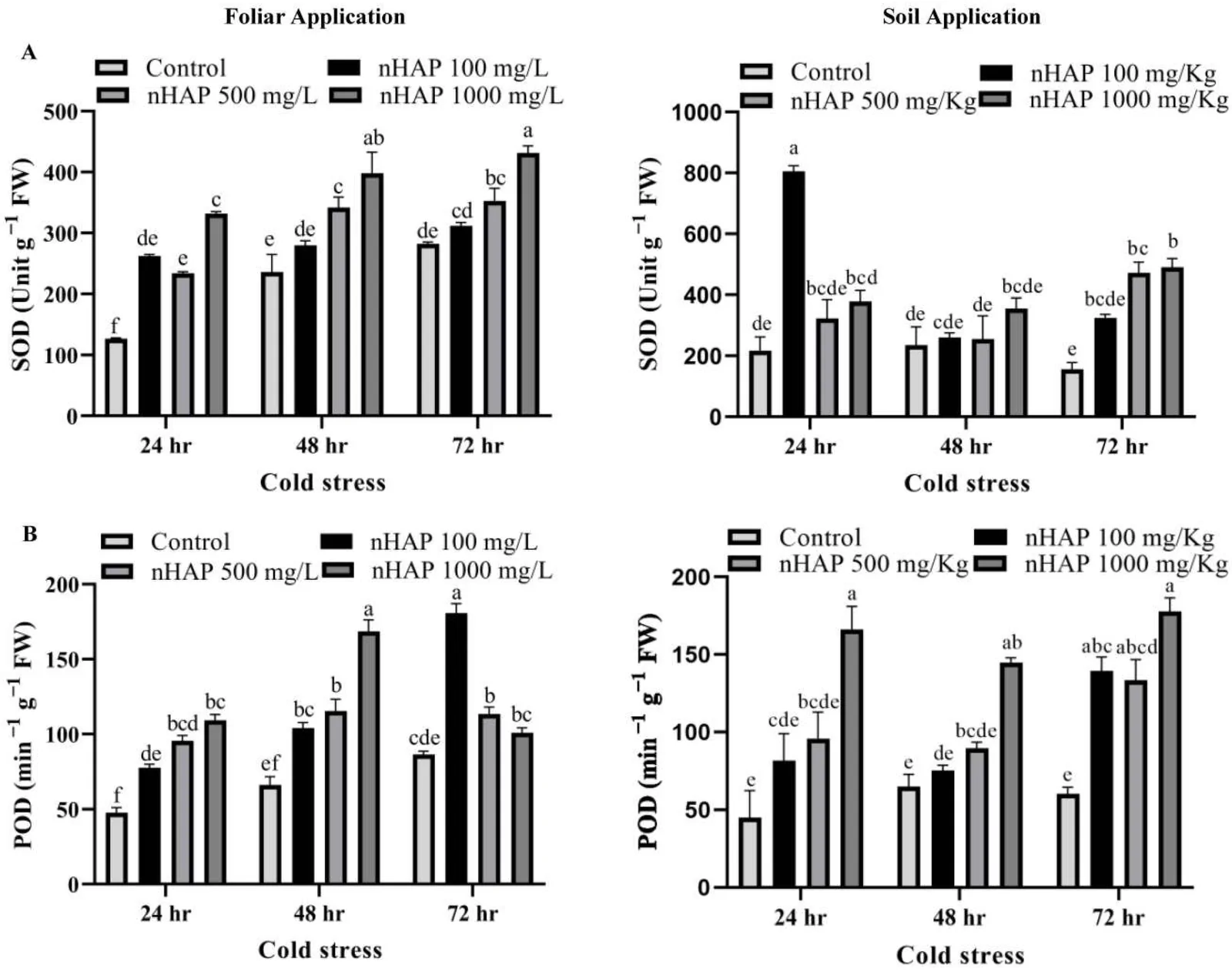
Effects of concentration and nHAP application method on antioxidant system during the cold treatment. (**A**) Superoxide dismutase (SOD). (**B**) Peroxidase (POD). Left, foliar Application; right, soil application. Measurements were taken at 24 h, 48 h and 72 h during the cold stress. Vertical bars show SEs of means (*n* = 3). Letters above bars denote significant differences among treatments (Tukey–Kramer HSD post hoc test with Benjamini–Hochberg FDR correction, *p* < 0.05).

Antioxidant enzyme activity was measured to assess the effect of nHAP (Figure 8). In control plants, SOD and POD activities increased gradually over time. Generally, nHAP-treated plants exhibited higher SOD and POD activity. In the foliar application, SOD activity increased by 161.53% with nHAP at 1000 mg/L at 24 h. At 48 h, the increase was 68.77% with the same treatment, and at 72 h it was 52.83% at 72 h compared to the control. In the soil application, SOD activity was significantly enhanced by 271.41% with nHAP 100 mg/kg at 24 h. At 48 h, there was a 50.34% increase, while at 72 h nHAP at 1000 mg/kg recorded a 214.51% increase in SOD activity. This suggests that prolonged stress benefits from higher nHAP concentrations for SOD activity in cucumber.

In the foliar application, POD activity (Figure 8B) in cucumber seedlings increased by 128.59% with nHAP at 1000 mg/L at 24 h, followed by a 154.33% increase at 48 h and a 16.50% increase at 72 h, indicating that higher nHAP concentrations consistently enhanced POD activity under cold stress. In the soil application, POD activity increased by 270.09% with nHAP at 1000 mg/kg during the first 24 h of cold stress, followed by a 122.33% increase at 48 h with the same treatment. At 72 h, POD activity increased by 195.50% compared to the control group, confirming the efficacy of nHAP in enhancing POD activity in cold-stressed cucumber seedlings.

### 2.7. Effect of nHAP on Gene Expression of CsCu/Zn SOD, CsMnSOD, CsSPX-MFS 1 and CsSPX-MFS 2

**Figure 9:**
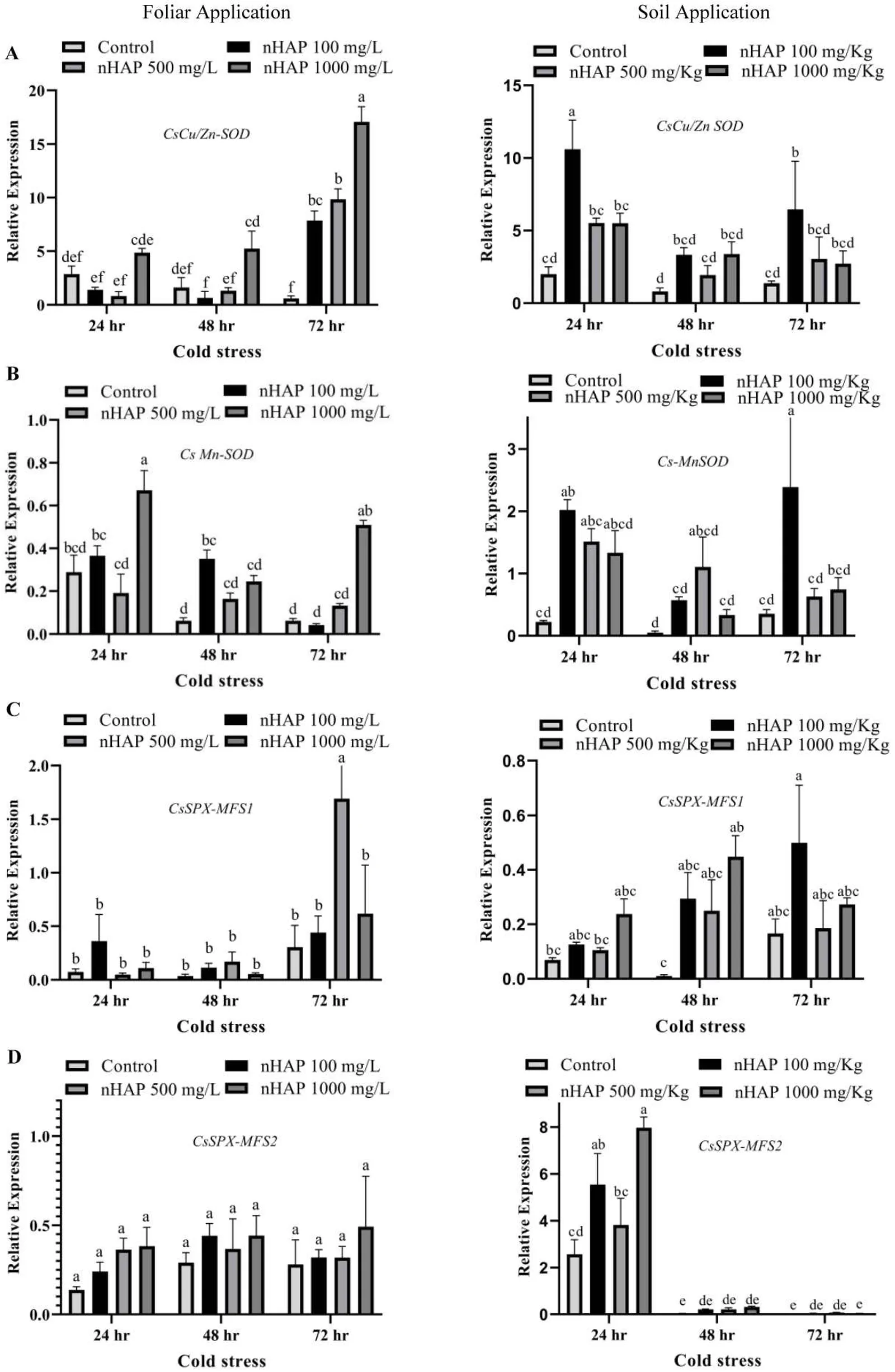
Effects of concentration and nHAP application method on expression levels of SOD-related genes during the cold treatment. (**A**) CsCu/ZnSOD. (**B**) CsMnSOD. (**C**) CsSPX-MFS1. (**D**) CsSPX-MFS2. Left, foliar application; right, soil application. Measurements were taken at 24 h, 48 h and 72 h during the cold stress. Vertical bars show the standard errors (SEs) of the means of 3 seedlings. The different letters on the columns indicate significant differences (*p* < 0.05) in Duncan’s multiple range test.

The relative mRNA expression of *CsCu/ZnSOD* (CsaV3_1G039510), Cs*MnSOD* (CsaV3_1G004740), *CsSPX-MFS1* (CsaV3_5G003300) and *CsSPX-MFS2* (CsaV3_6G005890) under cold treatment was determined by qRT-PCR (Figure 9). In control plants, the transcription levels of these genes generally decreased during the treatment, except for *CsSPX-MFS2*.

For the *CsCu/ZnSOD* gene (Figure 9A), foliar application of nHAP upregulated its expression level across all treatments and time points, with the highest expression observed at 24 h with 1000 mg/L. In the soil application, gene expression was highest at 24 h with nHAP at 100 mg/kg, while at 72 h and 48 h both 100 and 1000 mg/L showed higher expression.

For the *CsMnSOD* gene (Figure 9B), foliar application of nHAP upregulated the expression level at 24 h with 1000 mg/L, at 48 h across all concentrations, and at 72 h with 1000 mg/L. In the soil application, all the nHAP treatments upregulated its expression, particularly at 72 h with 100 mg/L.

For the *CsSPX-MFS*1 gene (Figure 9C), foliar application showed the strongest upregulation at 500 mg/L at 72 h in the cold treatment, while no significant effects were observed under the other treatments. In the soil application, nHAP significantly upregulated its expression at 24 h and 72 h with a concentration of 100 mg/L and at 48 h across all concentrations (100 mg/L, 500 mg/L and 1000 mg/L).

For the *CsSPX-MFS2* gene (Figure 9D), foliar application of nHAP had no significant influence on its expression level. However, soil application of nHAP increased its expression level at 24 h under cold treatment, although no significant influences were observed at 48 h or at 72 h.

### 2.8. Transcriptome Profiling of nHAP-Treated Seedlings by RNA-Seq

The transcriptome changes during the cold treatment were profiled by RNA-seq. There were three RNA-seq samples: CK (control: no nHAP and no cold stress), 0nHAPCold (no nHAP + cold stress), and 1000nHAPCold (1000 mg/L nHAP + cold stress). Each sample yielded a minimum of 8 GB of clean data, and a total of 95 GB of data were obtained across all samples. For each RNA-seq, at least 95.72% of bases achieved a Q30 quality score (Table S1).

**Figure 10:**
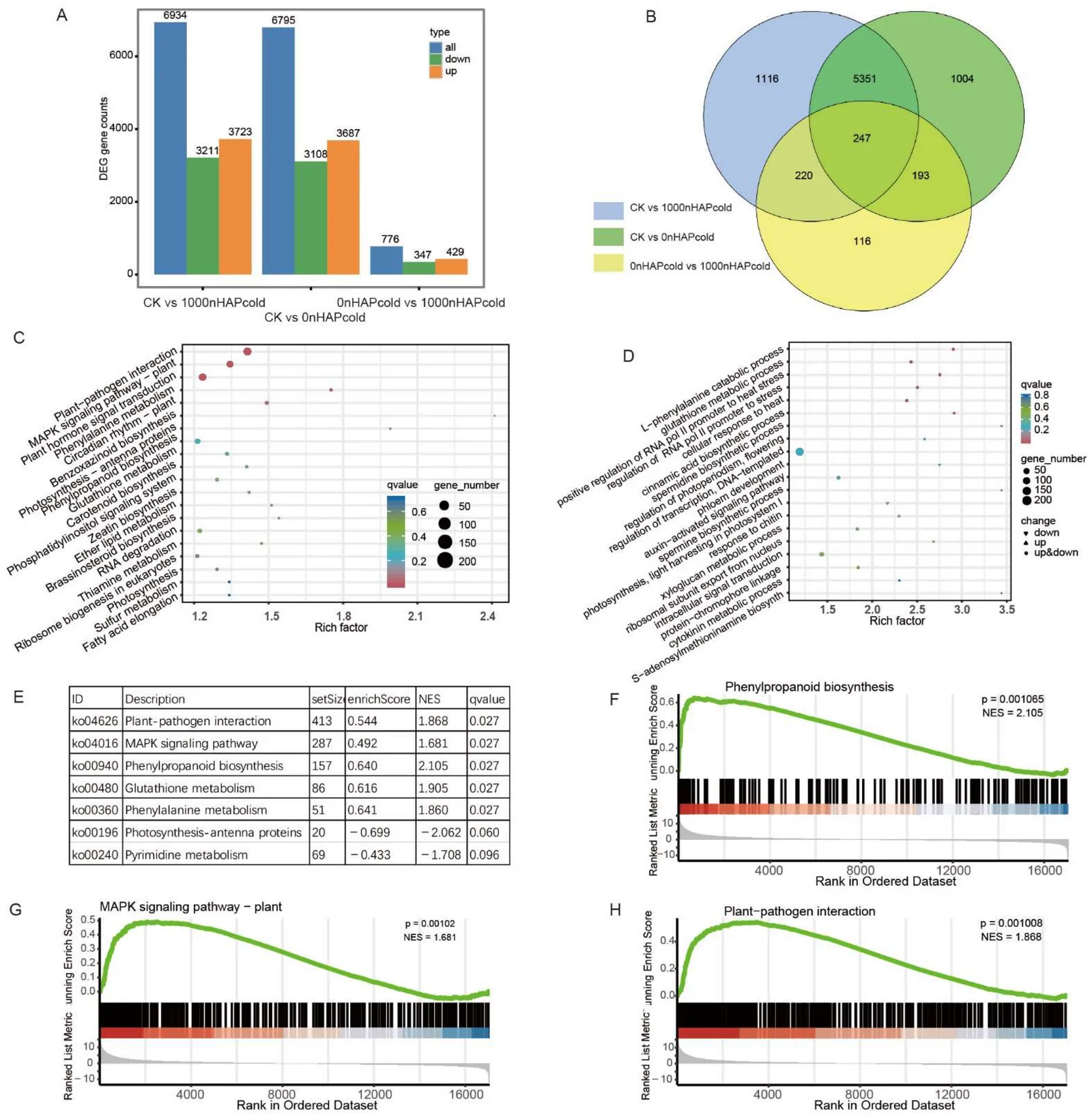
DEGs identified by RNA-seq among all treatment groups and DEGs identified during the cold treatment without nano-fertilizer application (CK vs 0 nHAPCold). (**A**) DEGs among different treatment groups. (**B**) Venn diagram of DEGs. (**C**) KEGG enrichment analysis displayed by dotplot. (**D**) GO enrichment analysis displayed by dotplot. (**E**) Significant enrichment by GSEA. (**F**–**H**) Plot of the top three most significant enriched pathways. Green curve: running enrichment score; black bars: gene positions in the ranked list; red-to-blue gradient: ranked list metric (red = upregulated, blue = downregulated).

A total of 6795 DEGs were identified in the CK vs. 0nHAPCold comparison, with 3687 upregulated and 3108 downregulated genes (Figure 10A). Without the protection of nHAP, plants suffered the most severe cold damage. The CK vs. 1000nHAPcold comparison identified total 6934 DEGs (3723 upregulated and 3211 downregulated). The 0nHAPcold vs. 1000nHAPcold comparison (nHAP-treated vs. untreated under cold stress) yielded far fewer DEGs—only 776 (429 upregulated and 347 downregulated). The difference in DEG number implies that the effect of cold stress overwhelmed that of the nHAP application. Similarly, Venn diagram analysis showed that only 247 DEGs were shared among all comparisons, whereas CK vs. 0nHAPCold and CK vs. 1000nHAPCold shared as many as 5598 DEGs (Figure 10B). Temperature is the predominant factor in affecting the plant transcriptome.

### 2.9. Profiling DEGs Under the Cold Treatment

**Figure 11:**
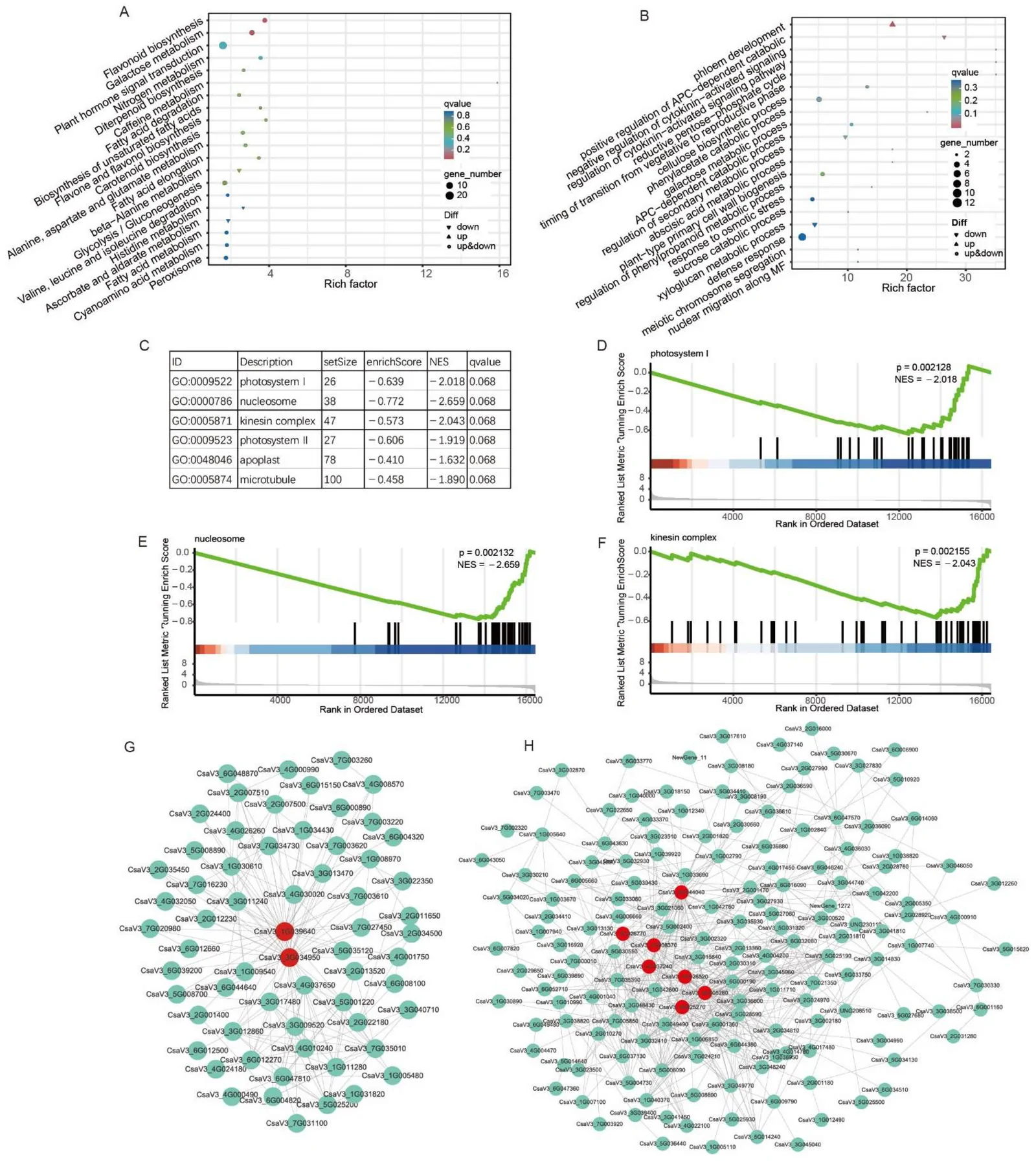
DEGs identified during the cold treatment between nHAP application and non-application (0 nHAPcold vs. 1000 nHAPCold). (**A**) KEGG enrichment analysis displayed by dotplot. (**B**) GO enrichment analysis displayed by dotplot. (**C**) Significant enrichment displayed by GSEA. (**D**–**F**) Plot of the top three most significant enriched pathways. Green curve: running enrichment score; black bars: gene positions in the ranked list; red-to-blue gradient: ranked list metric (red = upregulated, blue = downregulated). (**G**,**H**) Top two protein interaction networks of DEGs, displayed by cytoscape. Red nodes: hub genes; teal nodes: connected/co-expressed genes.

To profile DEG responses under cold stress without nHAP application, the enrichment of DEGs was analyzed for Ck vs. 0nHAPcold. All 6796 DEGs were enriched in 132 pathways by KEGG enrichment analysis (Figure 10C). The top three enriched pathways included plant–pathogen interaction (ko04626), phenylpropanoid biosynthesis (ko00940), and mitogen-activated protein kinase (MAPK) signaling pathway (ko04016). The GO enrichment analysis demonstrated that the DEGs were significantly enriched in regulation of “transcription, DNA templated”, “auxin-activated signaling pathway”, “intracellular signal transduction”, etc. (Figure 10D). Gene Set Enrichment Analysis (GSEA) (Figure 11C) revealed that plant–pathogen interaction (Normalized Enrichment Score (NES) = 1.868, q = 0.027) and phenylpropanoid biosynthesis (NES = 2.105, q = 0.027) are the most enriched pathways, along with MAPK signaling (NES = 1.681, q = 0.027) and glutathione metabolism (NES = 1.905, q = 0.027). On the other hand, photosynthesis—antenna proteins showed negative enrichment (NES = −2.062, q = 0.060) (Figure 10E). Enrichment plots from GSEA highlighted the top three enriched pathways with the highest positive NES values: plant–pathogen interaction (NES = 1.868), phenylpropanoid biosynthesis (NES = 2.105), and MAPK signaling (NES = 1.681) (Figure 10F–H).

### 2.10. Effects of nHAP Application on Cold-Stress Responses

To profile the transcriptomic differences between nHAP-treated plants and control plants under cold stress, functional enrichment analysis was performed for DEGs between 0nHAPcold and 1000nHAPcold. KEGG enrichment analysis of the DEGs reveals that a total of 776 DEGs were enriched in 105 pathways (Figure 11A). Plant hormone signal transduction (ko04075), galactose metabolism (ko00052), and glycolysis (ko00010) were the most enriched pathways, indicating that nHAP responds through the plant hormone signal transduction pathway under cold stress. GO enrichment analysis showed that DEGs were enriched in defense response and cellulose biosynthetic process (Figure 11B).

GSEA analysis showed the top five gene sets that demonstrate significant downregulation (negative NES values ranging from −1.89 to −2.65), including photosystem I/II, nucleosome, kinesin complex, and microtubule (Figure 11C). We plotted the GSEA enrichment for the top three enriched pathways with NES values: photosystem I (NES = 2.018), nucleosome (NES = 2.659), and kinesin complex (NES = 2.043) (Figure 11D–F). The analysis of the top protein–protein interaction (PPI) networks of DEGs shows that CsaV3_1G039640 (Acetyltransferase domain protein) and CsaV3_3G034950 (E3 ubiquitin-protein ligase XB3-like) position in the center of the network (Figure 11G). For one other PPI network, there are seven core genes, including CsaV3_6G044040 (BTB/POZ and TAZ domain protein), CsaV3_5G026770 (Protein FIZZY-RELATED like), CsaV3_4G008370 (transcription factor GTE4), CsaV3_4G037240 (Cyclin), CsaV3_5G026520 (Heat shock 70 kDa protein), CsaV3_3G005260 (Kinase family protein), and CsaV3_4G025270 (wall-associated receptor kinase-like 20) (Figure 11H).

### 2.11. nHAP Alleviates Cold Injury via Plant Hormone Signal Pathway

**Figure 12:**
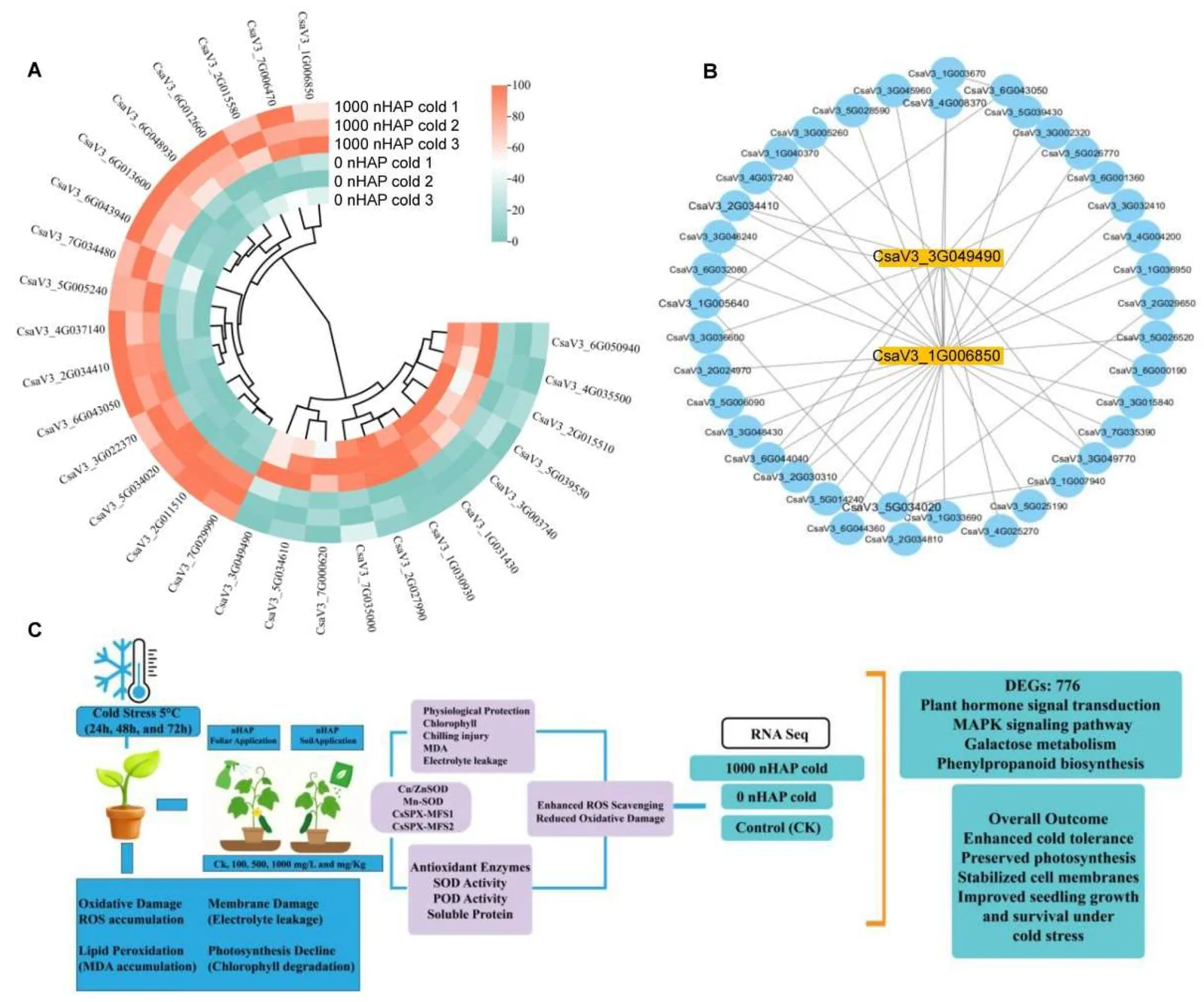
Plant hormone signal transduction pathway. (**A**) Heatmap of DEGs in the pathway. (**B**) PPI network of plant hormone signal transduction pathway. (**C**) Graphical demonstration of nHAP-induced cold-stress tolerance in cucumber plants.

The plant hormone signal transduction pathway was the most enriched pathway under nHAP treatment (0nHAPcold vs. 1000nHAPcold) with 28 DEGs. Response to cold stress under nHAP application was associated with upregulation of CsaV3_1G030930 (Auxin-responsive protein), CsaV3_2G015510 (SAUR24-like), CsaV3_6G012660 (GH3.6-like, maintaining auxin homeostasis) and CsaV3_2G015580 (SAUR21-like), showing the mechanism of nHAP in auxin-mediated growth and development (Figure 12A). Ethylene signaling was represented by CsaV3_3G022370 (an ERF/AP2 family member). The ERF/AP2 gene was upregulated under cold stress in the 1000nHAPcold group, and its expression was further increased by nHAP treatment, indicating a role for nHAP in enhancing ethylene-mediated cold-stress responses (Figure 12A), which are important for cell division and stress adaptation. A significant different in expression level was observed for Brassinosteroid-related CsaV3_2G027990 (BRI1 kinase inhibitor 1-like), the GA signaling repressor CsaV3_7G029990 (DELLA RGL2-like), and cytokinin-related CsaV3_2G034410 (APRR2) and CsaV3_3G049490 (APRR2) (Figure 12A).

The protein interaction of DEGs in the plant hormone pathway was demonstrated by PPI networks (Figure 12B). Two core hub genes were identified: CsaV3_1G006850 (receptor-like kinase, RLK) and CsaV3_3G049490 (Two-component response regulator-like protein APRR2). CsaV3_3G049490 (APRR2-like; log2FC = −1.203) interacted with 13 proteins, including CsaV3_2G034410 (APRR2), CsaV3_6G044040 (MYB transcription factor), and CsaV3_4G008370, implicating it in cytokinin/ABA-mediated transcriptional regulation. CsaV3_1G006850 (receptor-like kinase; log2FC = +1.556) was the most highly connected hub, interacting with 26 proteins, including MYB transcription factors (CsaV3_3G015840, CsaV3_3G048430), auxin-responsive protein CsaV3_5G026520, and CsaV3_7G035390, consistent with RLK roles as primary cold signal transducers activating downstream hormone cascades.

## 3. Discussion

This study demonstrates that nano-hydroxyapatite (nHAP) significantly enhances cold-stress tolerance in cucumber seedlings. Low temperature severely reduces photosynthetic efficiency, enzyme activity, and membrane integrity, making this protective mechanism essential [27] while nHAP was demonstrated to have the ability to mitigate these effects (Figure 12C).

The crystalline chemical compositions and crystal structures of nHAP and HAP are identical, and they have the same molecular formula. However, the nanoscale size effect results in different physical and chemical properties. Both HAP and nHAP showed statistically comparable effects on EL% reduction at all time points except for 24 h pre-treatment, which is consistent with the CII evaluation. The clear difference between nHAP and HAP emerges from LF-NMR and MRI data, where nHAP-treated seedlings maintained free water content (only ~12% LF-NMR T_2_ signal loss vs. ~50% in controls) and preserved tissue hydration with minimal necrotic damage in proton-density MRI images. The foregoing results reveal little disparity between nHAP and HAP in relieving chilling injury of cucumber seedlings, while nHAP has a distinct advantage in optimizing biological water activity under cold stress [27]. Although no plant mortality occurred, untreated control seedlings showed severe chilling injury approaching near-death phenotypes (extensive necrosis and loss of turgor), while nHAP-treated seedlings showed markedly reduced injury across all concentrations (Figure 2 and Figure 4). nHAP’s enhanced cold protection is likely due to its nanoscale size (TEM), which falls within the plant cell wall’s size-exclusion limit (~5–20 nm) and enables cellular uptake and enhanced water retention (LF-NMR/MRI) unavailable to bulk HAP, despite both sharing the same hydroxyapatite structure (FTIR) [28].

Foliar application of nHAP performed better than soil application in mitigating cold stress, which is mainly due to the immobilization of soil-applied nHAP. nHAP nanoparticles easily bind to clay minerals, Fe and Al oxides, and soil organic matter, limiting their mobility and drastically reducing the dissolution and release rate of P [25]. The particle size of HAPs negatively correlates with the efflux ratio. Smaller nHAP particles can migrate deeper or even leach away [29]. nHAP can be applied as droplets to leaf surfaces and enter through the stomata and diffuse through the apoplasts, where it is dissolved and releases P without entering the mesophyll cells [30]. For the soil column experiments [31], nHAP showed significantly lower mobility than traditional soluble phosphorus fertilizers, leading to significantly lower P leaching losses. We acknowledge soil application as a potentially complementary long-term strategy rather than an acute-stress mitigation tool.

When evaluating and comparing the fertilization efficiency of nHAP at various concentrations, 100 mg/L in foliar spray optimally reduced the chilling injury index. This is likely due to the complex and often biphasic nature of nanoparticle effects. Nanoparticles are characterized by low-dose stimulation and high-dose modulation of different physiological traits, which is called a hormetic response [32]. Additionally, we detected significant differences across multiple physiological indicators. For example, a concentration of 1000 mg/L instead of 100 mg/L performed the best in terms of EL% and SOD gene expression. This phenomenon of nHAP application has also been reported in cold-tolerance research on rice seedlings [33]. Lower doses likely facilitate more efficient stomatal uptake and signaling activation without saturation of foliar entry points, whereas higher concentrations enable greater phosphorus and calcium delivery, supporting membrane stability and antioxidant defense [30].

Cold stress is known to produce ROS, which cause oxidative damage to cell membranes, proteins, and lipids [34,35]. Both NBT and DAB staining assays clearly demonstrated that foliar spraying of 100 mg/L nHAP markedly reduced ROS accumulation. In contrast, soil application achieved optimal effects at the concentration of 1000 mg/L. These observations were consistent with the CII index measurements. Although the cold tolerance effects across various nHAP concentrations were inconsistent with other antioxidant-related physiological indices, including MDA, EL, SOD, and POD, the results distinctly verified that nHAP application effectively lowered MDA content and elevated the activities of SOD and POD in cucumber seedlings during the cold treatment. These results are consistent with previous studies about the application of nano-fertilizers (HP-NPs and ZnO-NPs) in the context of cold tolerance of plants [36]. Elevated EL and MDA are established indicators of cellular damage and increased membrane permeability [37]. The enhancement in SOD and POD activities is associated with improved ROS scavenging capacity and reduced oxidative damage [38]. Increased SOD and POD activity could theoretically reflect nanoparticle-induced stress rather than adaptation, as dose-dependent phytotoxicity and genotoxicity of hydroxyapatite nanoparticles have been reported in other species [39]. However, MDA and electrolyte leakage in nHAP-treated plants decreased toward healthy control levels, alongside improved chlorophyll retention, favoring an adaptive rather than toxic response. Growth/biomass and genotoxicity were not assessed here and should be addressed in future work.

A comparable increase in SOD and POD was reported for the application of nHAP in a cold experiment on *Brassica chinensis* [19], nano-silicon treatments in cold-stressed tomato [40], and nHAP and iron oxide (Fe_3_O_4_) nanoparticles in a cadmium-stress experiment on rice seedlings [41]. The elevation in SOD and POD activities induced by nHAP was confirmed to be associated with the upregulation of their gene expression levels. Lower expression of *CsCu/ZnSOD* and *CsMnSOD*, which are involved in ROS detoxification and related to Mn^2+^, Cu^2+^, or Zn^2+^ concentrations, resulted in ROS level elevation [42]. Our study indicates that foliar spray of nHAP with a concentration of 1000 mg/L significantly upregulated the expression of *CsCu/ZnSOD* after 24 h and 72 h in cold stress, while in the soil application the concentration of 100 mg/Kg performed the best (Figure 9).

Here we speculate that nHAP may provide bioavailable P and Ca, which in turn enhance energy metabolism, regulate membranes, support normal function of chloroplasts, and finally reduce ROS in cold stress [41]. Although P and Ca were not detected in cucumber leaves here, the bioavailability and uptake of nHAP-derived phosphorus and calcium are well documented in other publications. nHAP releases bioavailable phosphate and Ca^2+^ ions that support energy metabolism, membrane stability, and cold-stress signaling [43,44,45]. SOD activity increase, MDA content decrease, and membrane integrity improvement are involved in the established roles of P in ATP and membrane lipids and Ca^2+^ as a second messenger in ROS modulation and cold-tolerance pathways [46,47,48]. We therefore propose that nHAP functions as a slow-release source of P and Ca under chilling stress. Direct tissue quantification remains a priority for future work.

The plant hormone signal transduction pathway seems to play a major role in nHAP-induced cold tolerance, which is revealed by comparative transcriptomic analysis. nHAP application with a concentration of 1000 mg/L in the cold treatment (1000nHAPcold) induced different expression of genes encoding signaling components and receptors within the auxin, cytokinin, BR, GA, and ethylene pathways, as well as stress signaling pathways. The PPI network of DEGs in the plant hormone signal transduction pathway indicates the central role of *CsaV3_1G006850 (RLK)*, which belongs to a large superfamily and play roles in plant responses to multiple environmental stimuli [49]. *RLK* contains a highly variable extracellular domain for ligand binding and a cytoplasmic kinase domain for signal initiation, and it plays an important role in regulating plant growth, reproduction, stomatal opening and closure, hormone signal transduction, and plant adaptation to biotic and abiotic stresses [50]. The reduced DEG number under the nHAP treatment, together with the associated improvements in CII, EL, MDA, and chlorophyll content, suggests a smaller stress burden and a more targeted transcriptional response rather than suppression of the stress response, consistent with reports linking fewer stress-induced DEGs to greater genotypic resilience [51].

## 4. Materials and Methods

### 4.1. Plant Materials

Cucumber XIS49 is a sequencing material [5]. Seeds were soaked at 55 °C for 15 min in a hot water bath to facilitate uniform germination. They were then spread out on filter paper in Petri dishes for 24 h to germinate in the dark. Seeds with uniform germination were selected and sown in trays containing a mixture of peat, vermiculite, and perlite in a 2:1:1 ratio. Growing conditions were maintained at 25 °C, with a 12 h light/dark cycle and a light intensity of 600 µmol·m^−2^·s^−1^ in plant growth incubators (GTOP-310Y, Zhejiang Top Instrument Co., Ltd., Hangzhou, Zhejiang, China). When the seedlings reached 3–4 true leaves, uniform seedlings were selected for the experiment.

### 4.2. Assessment of Chilling Injury

To assess cold-induced injury, the chilling injury index (CII) was measured using the method described by [52]. Chilling injury severity was classified into five levels, with scores assigned as follows: score 0 indicates no symptoms of injury, score 1 represents very slight cold injury equivalent to 5% of the leaf surface, score 2 is attributed to 5–20% leaf surface injury, score 3 indicates moderate injury covering up to 50% of the leaf surface, score 4 demonstrates serious injury affecting more than 50% of the leaf surface, and score 5 indicates a dead plant. After scoring the plants according to the above criteria, the CII was calculated using the following formula:

CII = (∑Corresponding Grade × Number of Plants of per Grade)/(Number of Total Plants × Highest Grade) × 100%

### 4.3. Preparation of nHAP

The selected nHAP was synthesized using the wet chemical precipitation method [53]. In detail, solutions of 0.2 M Ca(NO_3_)_2_, 0.12 M NaH_2_PO_4_, and 0.28 M NaOH were prepared separately. Then, 250 mL quantities of the Ca(NO_3_)_2_ and NaH_2_PO_4_ solutions were added simultaneously and dropwise to a beaker containing 500 mL of NaOH solution at the same rate over 2 h, with vigorous stirring throughout the process. The mixture was aged at room temperature for 12 h and was then allowed to settle overnight to precipitate the hydroxyapatite nanoparticles. Finally, the nHAP sample was washed with ethanol and deionized water to remove excess ions, then vacuum-dried and sieved by a vacuum drying oven (DZF-6050, Shanghai Yiheng Scientific Instrument Co., Ltd., Shanghai, China). nHAP was stored in vacuum glass bottles on room temperature.

### 4.4. nHAP Application Under Cold Treatment and Sample Preparation

To compare the effects of nHAP and HAP, seedlings at the 3–4 leaf stage were foliar-sprayed with nHAP and HAP at 1000 mg/L at 8 h, 16 h, 24 h, and 48 h before cold-stress treatment. After nHAP treatment, the seedlings were then exposed to cold treatment in in plant growth incubators (GTOP-310Y, Zhejiang Top Instrument Co., Ltd., Hangzhou, Zhejiang, China) at 5 °C, with samples collected at 0 h, 12 h, and 24 h after treatment initiation. Cold treatment conditions were 5 °C with a 12 h light/dark cycle and a light intensity of 600 µmol·m^−2^·s^−1^. CII values were investigated, and fresh samples were subjected to electrolyte leakage (EL) assay. Three seedlings were included per sample to serve as biological replicates, and each sample was tested in triplicate as a technical replicate.

For biological water detection, foliar sprays of nHAP and HAP at a concentration of 1000 mg/L were applied 24 h before cold-stress treatment. Fresh leaves were collected at 0 h and 24 h during the cold treatment and were then subjected to biological water detection by low-field nuclear magnetic resonance (LF-NMR) and magnetic resonance imaging (MRI) assays.

We optimized nHAP fertilization methods (foliar spray and soil application), application time (24 h, 48 h, and 72 h before cold treatment), and concentration (100 mg/L, 500 mg/L, and 1000 mg/L). For foliar application, nHAP suspensions were freshly prepared at concentrations of 100, 500, and 1000 mg/L, plus a control (Ck), and magnetically stirred for 10 min to ensure uniform dispersion and used immediately. Tween-20 was added as a surfactant to prevent particle settling. Seedlings were sprayed until fully wet with run-off. For soil application, nHAP powder was mixed into soil at doses of 100, 500, and 1000 mg/kg, plus control soil. nHAP powder was weighed and thoroughly mixed with 1 kg soil in a clean container, then transferred to pots. Control seedlings were treated with HAP or non-HAP/nHAP. CII values were investigated during the treatment. Fresh leaf samples were used to detect EL. Leaf samples stored under ultra-low temperature at −80 °C were used to detect MDA content, soluble protein content, chlorophyll content, carotenoid content, ROS accumulation, SOD and POD content, as well as gene expression of CsZu/ZnSOD, CsMnPOD, CsSPX-MFS1, and CsSPX-MFS2. Three seedlings were included per sample to serve as biological replicates, and each sample was tested in triplicate as a technical replicate.

For RNA-seq, leaf samples from seedlings were collected at normal temperature without any nHAP or cold treatment (CK), with no nHAP application at 24 h after cold treatment (0nHAPCold), and with nHAP application at a concentration of 1000 mg/L before 24 h of the cold treatment and sample collection at 24 h after the cold treatment (1000nHAPCold). Each RNA-seq sample contained 3 independent biological replicates.

### 4.5. Electrolyte Leakage (EL) and Malondialdehyde (MDA) Content

The biggest leaf of each seedling was used to measure EL by an electrical conductivity meter (DDS-307A, INESA Scientific Instrument Co., Ltd., Shanghai, China). A leaf disk weighing 0.20 g was immersed in 10 mL of deionized water and the initial electrical conductivity (*EC1*) was measured. After heating the tubes at 100 °C for 30 min, the final electrical conductivity (*EC2*) was assessed. The method followed an open publication [54]. EL was calculated using the following equation:

EL% = *EC*_1_/*EC*_2_ × 100%

The variables *EC*_1_ and *EC*_2_ denote the initial conductivity and the conductivity after the water bath.

MDA content was measured to assess oxidative damage caused by cold stress, as MDA is an indicator of lipid peroxidation and membrane damage. MDA levels were determined using thiobarbituric acid (TBA) [55].

### 4.6. Determination of Antioxidant Enzyme Activities

The three largest leaves of each seedling were used to measure antioxidant enzyme activities, and the detection method was as described in an open publication [56]. To estimate the activity of SOD and POD, fresh leaf samples (0.2 g) were ground on ice in 2 mL of ice-cold phosphate buffer (0.05 M, pH 7.8). The homogenate was transferred to a centrifuge tube, and the volume was adjusted to 5 mL with the same buffer. The mixture was then centrifuged at 12,000 rpm for 20 min at 4 °C to obtain a clear supernatant, which served as the enzyme extract.

SOD activity was determined by the nitroblue tetrazolium (NBT) method [57,58]. The reaction mixture was prepared using NBT, methionine, riboflavin, EDTA-Na2, and distilled water (pH 7.8), and 30 μL of enzyme extract was added to 3 mL of this solution. The mixture was placed under fluorescent light at 4000 lux for 30 min, while control samples were kept in the dark. Absorbance was quantified at 560 nm by a UV–Vis spectrophotometer (Thermo Fisher Scientific, Waltham, MA, USA). For POD activity, the reaction mixture consisted of 0.1 M phosphate buffer (pH 6.0), guaiacol, and 30% H_2_O_2_. 100 μL of enzyme extract was added to 3 mL of reaction solution. The absorbance at 470 nm was recorded by a UV–Vis spectrophotometer (Thermo Fisher Scientific, Waltham, MA, USA) at 30 s intervals over 3 min.

### 4.7. Histochemical Assay of O_2_^−^ and H_2_O_2_

The three largest leaves of each seedling were used. O_2_^−^ and H_2_O_2_ were detected through histochemical staining using NBT and 3,3′-diaminobenzidine (DAB), respectively, as described by [59]. Fresh leaf samples were submerged in freshly prepared 1 mg/mL NBT solution (pH 7.8) and DAB solution (pH 3.8) at room temperature in the dark for 6–8 h. The stained leaves were first washed with distilled water, then immersed in 95% ethanol and kept in a water bath at 75 °C for 15–20 min for decolorization.

### 4.8. Chlorophyll Content and Total Soluble Protein

The three largest leaves of each seedling were used. Chlorophyll content was estimated using a 95% ethanol solution in 10 mL tubes, with each tube containing 0.20 g of fresh leaf sample, as described by [60]. The tubes were kept in the dark for 16–24 h until the leaves turned white. Chlorophyll content was measured at 470 nm, 649 nm, and 665 nm by using a UV–Vis spectrophotometer (Thermo Fisher Scientific, Waltham, MA, USA). Soluble protein was measured using Coomassie brilliant blue G-250 staining, as described by [61].

### 4.9. LF-NMR and MRI Measurements

LF-NMR measurements were conducted using a Niumag NMR system (NMR20-060H-I, Suzhou Niumag Analytical Instrument Corporation, Suzhou, China) equipped with a 60 mm radiofrequency coil (NMR20-060H-I-60mm). T2 relaxation times were acquired using a Carr–Purcell–Meiboom–Gill pulse sequence with the following parameters: spectrometer frequency = 21 MHz, transmitter frequency offset = 298,554.72 Hz, 90° pulse width = 14.00 μs, 180° pulse width = 27.04 μs, echo time = 0.200 ms, number of echoes = 18000, spectral width = 200 kHz, receiver dead time = 0.002 ms, and number of scans = 4. The receiver gain was set to 20.0 dB, with phase-related gain = 1 and digital receiver gain = 3. Waiting time was set to 4500 ms between acquisitions. T2 relaxation data were processed and analyzed to assess water mobility and distribution changes in cucumber seedlings subjected to cold-stress treatment, and the results were analyzed using standard exponential decay fitting procedures [62].

MRI measurements were performed according to [63] using a Niumag NMR imaging system V3.0 (Niumag Corporation, Shanghai, China) equipped with a 60 mm radiofrequency coil. The top newly developed leaves of similar size from each treatment were used. T2-weighted images were acquired using a Hahn spin-echo pulse sequence with the following imaging parameters: repetition time = 3750 ms, echo time = 20 ms, flip angle = 90°, refocus flip angle = 180°, read size = 256 pixels, phase size = 192 pixels, and echo position = 50%. Radiofrequency gain was set to 20.0 dB, phase-related gain to 3, and digital receiver gain to 2 dB. Additional acquisition parameters included a phase encoding duration of 3.00 ms and a sweep width of 20 kHz. No dummy scans were applied before data acquisition.

### 4.10. RNA Extraction and Quantitative Real Time-PCR (qRT-PCR)

The transcription levels of *CsCu/ZnSOD* (CsaV3_1G039510), *CsMnSOD* (CsaV3_1G004740), *CsSPX-MFS1* (CsaV3_5G003300), and *CsSPX-MFS2* (CsaV3_6G005890) were quantified by qRT-PCR. Fresh leaf samples were collected in liquid nitrogen and stored at −80 °C for RNA extraction. Total RNA was extracted from all leaf samples using RNAiso Plus (Takara Biotechnology (Dalian) Co., Ltd., Dalian, China) according to the manufacturer’s instructions. First-strand cDNA synthesis was performed using the PrimeScript™ RT Reagent Kit with gDNA Eraser (Takara Biotechnology (Dalian) Co., Ltd., Dalian, China) according to the manufacturer’s instructions.

The cDNA was diluted 5-fold and used as the template for amplification. Real-time PCR was performed using TB Green^®^ Premix Ex Taq™ II (Tli RNaseH Plus, Cat. No. RR820A; Takara Biotechnology (Dalian) Co., Ltd., Dalian, Liaoning, China) on an StepOne™ Real-Time PCR System (Applied Biosystems, Foster City, CA, USA) to detect fluorescence amplification. CsUbiquitin were used as reference genes. Relative mRNA expression levels of the target genes under different nHAP treatments and cold-stress durations were calculated using the 2^−ΔΔCt^ method [64]. The primers used in this study are listed in Table S2.

### 4.11. RNA-Seq

After total RNA extraction, the purity, concentration, and integrity of the RNA samples were assessed prior to transcriptome sequencing analysis using an Illumina HiSeq2500 high-throughput sequencing platform (Illumina Inc., San Diego, CA, USA). Clean reads were aligned to the *Cucumis sativus* ChineseLong v3 reference genome (http://cucurbitgenomics.org/organism/20 (accessed on 11 May 2025) using HISAT2 (version 2.2.2) [65], with average mapping ratios ranging from 95.30% to 96.85% across samples. Gene expression levels were quantified using the fragments per kilobase of transcript per million mapped reads (FPKM) method. Differential expression analysis between samples was performed using DESeq2 (version 1.48.2), with genes identified as differentially expressed based on a Benjamini–Hochberg adjusted *p*-value (FDR) and a fold change (FC) ≥ 2. Finally, Gene Ontology (GO) functional annotation and Kyoto Encyclopedia of Genes and Genomes (KEGG) pathway enrichment analysis of the DEGs were performed.

### 4.12. Statistical Analysis

Data were subjected to one-way analysis of variance (ANOVA). Mean separation was performed using the Tukey–Kramer post hoc test, and *p*-values were further adjusted using Benjamini–Hochberg false discovery rate (FDR) correction for multiple comparisons. Means with different letters are significantly different at *p* < 0.05. All statistical analyses were performed in R (version 2026.05.0+218) using the emmeans and multcompView packages.

## 5. Conclusions

nHAP application effectively alleviated chilling injury in cold-sensitive XIS cucumber seedlings through coordinated physiological and molecular mechanisms. Foliar application consistently outperformed soil application, with 1000 mg/L identified as the optimal concentration. nHAP preserved both free and bound water status during cold exposure and enhanced chilling tolerance in cucumber primarily by reinforcing antioxidant defense and modulating hormone signaling pathways.

## Data Availability

The original contributions presented in this study are included in the article/Supplementary Materials including Figure S1. Further inquiries can be directed to the corresponding author.

## Supplementary Materials

The following supporting information can be downloaded at https://www.mdpi.com/article/10.3390/ijms27167358/s1.
