# Supplementary material for: Physiological and Molecular Mechanisms of Nano-Hydroxyapatite (nHAP) in Regulating Chilling Tolerance of Cucumber Seedlings
Source: Int J Mol Sci. 2026 Aug 17;27(16):7358. doi: 10.3390/ijms27167358 (PMC13513064; doi:10.3390/ijms27167358)
Supplement: Supplementary file 1 [file ijms-27-07358-s001.zip › ijms-4459838-supplementary.pdf]

Table S1. Statistics of RNA quality sequencing data.

SampleID	ReadSum	BaseSum	GC(%)	N(%)	Q20(%)	Q30(%)
CK-1	24777996	7.38E+09	44.76	0	100	95.59
CK-2	25255197	7.53E+09	44.76	0	100	95.62
CK-3	22707754	6.78E+09	44.76	0	100	95.72
H1000-1	22485477	6.72E+09	43.69	0	100	96.11
H1000-2	23682842	7.06E+09	43.66	0	100	95.79
H1000-3	25860482	7.71E+09	43.48	0	100	95.58
HZ-1	26155967	7.82E+09	43.64	0	100	95.47
HZ-2	20268494	6.06E+09	43.72	0	100	95.81
HZ-3	24671576	7.38E+09	43.72	0	100	95.64

Table S2. Primer sequences for qRT-PCR.

Primers used in this study		Sequence (5′→3′)
Gene Name	F/R Primer	
CsCu/ZnSOD	F	GACTGGGCCACA TTTCAACC
CsCu/ZnSOD	R	GCCTTGCCATCTTCACCAA
CsMnSOD	F	CAATGGCGG AGGTCACATTA
CsMnSOD	R	AGAGCAAGCCACA CCCATC
CsSPX-MFS1	F	CCTGGTTGGGTTATGGCTGT
CsSPX-MFS1	R	AGGTCACTGGTAGGCGAGAT
CsSPX-MFS2	F	GTGCTCGGCTCTAATCACATTTG
CsSPX-MFS2	R	GTTTACTTTCACTCAAGTACCC
CsaV3_4G005460	F	CTCCTCGGAAGATGATGGGC
CsaV3_4G005460	R	CGTATAACCTCCCGGTGTCG
CsaV3_2G007510	F	TAGTGCAGCAGCCTTTGTTG
CsaV3_2G007510	R	ATGGACGAGGCAATGTCAGG
CsaV3_5G035120	F	AGGAGGCCTCTTCAGCCTTA
CsaV3_5G035120	R	AAAGCAGTGGCAAACGCAAA
CsaV3_4G008080	F	GGAAGAGAGGGATTGGCAGG
CsaV3_4G008080	R	GAGAGTGGCCATGCCATAGA
CsaV3_7G013790	F	CAAGTGGTGGCTGGAACCAA
CsaV3_7G013790	R	TGAAGAATGTGAGCTGCTTGA

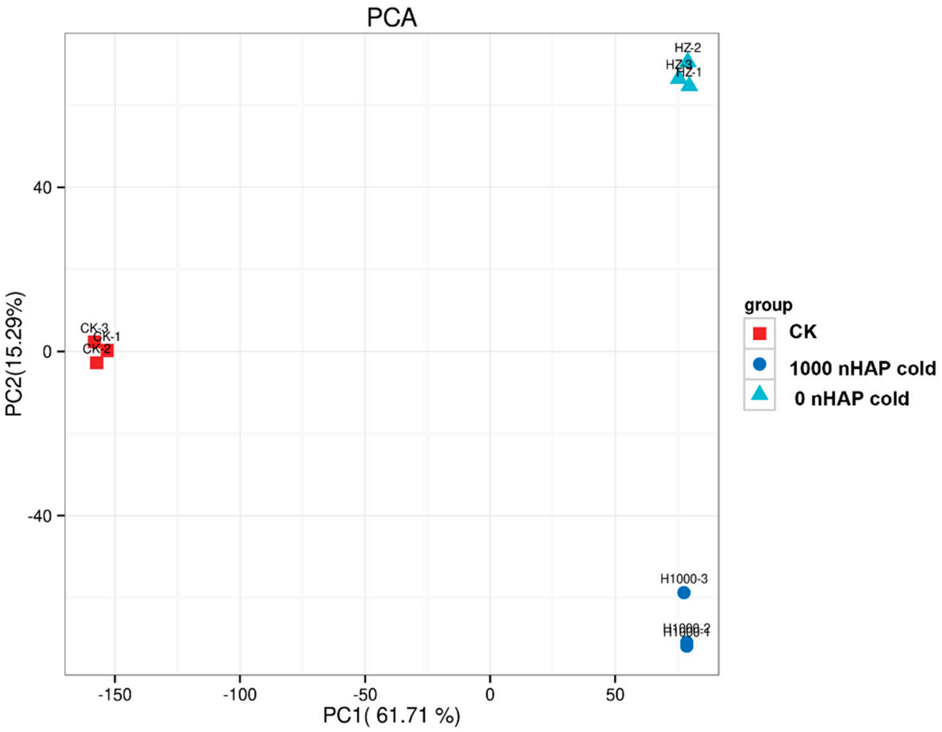

Figure S1. Correlation assessment of biological replicates. PCA plot showing the distribution of biological replicates across three experimental groups: CK (red squares), 1000 nHAP cold (blue circles), and 0 nHAP cold (cyan triangles). PC1 and PC2 account for 61.71% and 15.29% of the total variance, respectively, demonstrating clear inter-group separation and high intra-group reproducibility.
